# NoD: a Nucleolar localization sequence detector for eukaryotic and viral proteins

**DOI:** 10.1186/1471-2105-12-317

**Published:** 2011-08-03

**Authors:** Michelle S Scott, Peter V Troshin, Geoffrey J Barton

**Affiliations:** 1 Division of Biological Chemistry and Drug Discovery, College of Life Sciences, University of Dundee, Dow Street, Dundee DD1 5EH, UK

**Keywords:** nucleolus, protein targeting signal, protein localization, NoD web server

## Abstract

**Background:**

Nucleolar localization sequences (NoLSs) are short targeting sequences responsible for the localization of proteins to the nucleolus. Given the large number of proteins experimentally detected in the nucleolus and the central role of this subnuclear compartment in the cell, NoLSs are likely to be important regulatory elements controlling cellular traffic. Although many proteins have been reported to contain NoLSs, the systematic characterization of this group of targeting motifs has only recently been carried out.

**Results:**

Here, we describe NoD, a web server and a command line program that predicts the presence of NoLSs in proteins. Using the web server, users can submit protein sequences through the NoD input form and are provided with a graphical output of the NoLS score as a function of protein position. While the web server is most convenient for making prediction for just a few proteins, the command line version of NoD can return predictions for complete proteomes. NoD is based on our recently described human-trained artificial neural network predictor. Through stringent independent testing of the predictor using available experimentally validated NoLS-containing eukaryotic and viral proteins, the NoD sensitivity and positive predictive value were estimated to be 71% and 79% respectively.

**Conclusions:**

NoD is the first tool to provide predictions of nucleolar localization sequences in diverse eukaryotes and viruses. NoD can be run interactively online at http://www.compbio.dundee.ac.uk/nod or downloaded to use locally.

## Background

The nucleolus is a sub-nuclear cellular compartment that is accessible to a large number of proteins since it is not surrounded by a membrane. To date, over 4500 distinct human proteins have been identified from purified nucleoli [1]. The most well-characterized function of the nucleolus is the biogenesis of ribosomes [2]. However, nucleolar proteins are diverse and dynamic, reflecting the central role of this compartment in the cell through its involvement in numerous other key cellular processes and in the cellular response to changing conditions [3-7]. Indeed, many proteins have been found to localize cyclically or conditionally to the nucleolus [3,4,7,8].

Although such a large and dynamic volume of cellular traffic likely requires extensive regulation, proteins are often proposed to localize to the nucleolus simply through high-affinity binding to core nucleolar components [6,9]. Despite this, numerous disparate reports of short nucleolar targeting sequences in proteins have been published over the past 20 years. Many of these sequences can localize non-nucleolar reporter proteins to the nucleolus when fused to them. In an effort to catalogue and systematically characterize these Nucleolar Localization Sequences (NoLSs), we have recently curated the literature and assembled a human NoLS dataset which we subsequently used to train an artificial neural network computational predictor [10]. The predictor considers the protein sequence and JPred predictions of protein secondary structure [11]. When applied to the entire human proteome, it identified thousands of candidate NoLSs, ten of which were experimentally tested and confirmed to target the nucleolus [10].

Here, we describe NoD, a web server and a command-line program that provides computer predictions of NoLSs in proteins. We also investigate the application of the human-trained predictor in other eukaryotic and viral organisms, demonstrating that NoD can give effective NoLS predictions in a wide variety of species.

### Implementation

**Figure 1:**
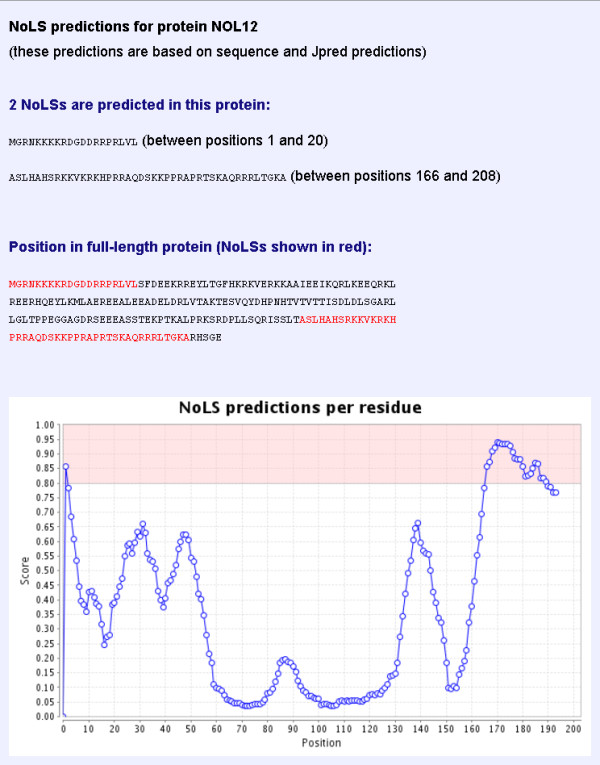
**Example of NoLS prediction returned by NoD**. If at least one NoLS is predicted in a protein, NoD returns an output page that displays the sequence and position of the predicted NoLSs, the full-length protein sequence as entered by the user with the NoLSs in red and a graph showing the average NoLS prediction score for every 20-residue window in the protein. The region shown in pink in this graph is the NoLS candidate segment region and represents the range of scores within which a 20-residue segment is predicted to be a NoLS.

The NoD web server provides an easy way to predict NoLSs within a protein sequence. NoD predictions are obtained by entering a protein sequence in fasta format on the NoD webserver http://www.compbio.dundee.ac.uk/nod. Protein sequences are encoded as previously described [10]. Briefly, sliding windows of size 13 are sparsely encoded in a binary format using a reduced alphabet of size 12 for submission to an artificial neural network (ANN). The current implementation of NoD uses a local version of Batchman from the Stuttgart Neural Network Simulator [12] and the human-trained NoLS prediction model developed previously [10] to provide the prediction for each encoded subsequence. The Batchman output is then processed and NoLSs are predicted if the average score output by the ANN of 8 consecutive windows is at least 0.8 [10]. Finally, the prediction is displayed as shown in Figure 1 if at least one NoLS is identified. Otherwise, the user is informed that no NoLS is predicted in the input protein. As shown in Figure 1, for proteins predicted to contain NoLS(s), the output consists of 3 sections:

- the sequences of the predicted NoLS(s) are first enumerated

- the full-length protein sequence is displayed with the predicted NoLS(s) shown in red

- finally, a graph is presented of the NoLS window-based score [10] as a function of position in the protein sequence.

The NoLS window-based score graph can be useful to guide experimental design of nucleolar targeting. The graph gives an overview of the entire protein and shows the proportion of the protein with putative nucleolar targeting capabilities as well as regions of the protein that are near the cut-off threshold and therefore almost predicted as NoLSs.

When entering a protein sequence, the user is provided with the option of also running JPred secondary structure predictions [11] to include as input to the NoLS neural network. If JPred is selected, the accuracy of prediction is slightly higher [10] but the computation time is increased.

For users who want predictions for whole proteomes there is a command line version of NoD called clinod. Clinod produces the same results as a web server but it is more suitable for processing of multiple sequences and is convenient to use within software pipelines.

Clinod requires Java 6 and the Batchman executable from the Stuttgart Neural Network Simulator [12] to run. Clinod accepts the list of FASTA formatted sequences from an input file and outputs the predictions to a file or the console. By default the following output is produced for each sequence-the name of the sequence, the number of NoLSs predicted, the start and the end positions and the sequences of each predicted NoLS. However, for better integration with other bioinformatics tools, many more output options are supported. For example, the input sequences can be cleaned (stripped of ambiguous characters), and output along with the prediction results and sequences with no predicted NoLS can be omitted from the output. Various output options are described in Table 1 but for a detailed description of the clinod switches please refer to Additional file 1.

**Table 1:** Clinod output formats

Format name	Format Description	Example output^a^
MINIMAL	Sequence name and number of NOLS predicted	> NOL12 NOLS_segment_number: 2

SHORT	Same as MINIMAL plus, start and end position of each NOLS	> NOL12 NOLS_segment_number: 2 NOLS_segments_positions: 1-20, 165-213

MEDIUM (default)	Same as SHORT plus the sequences of all NOLS	> NOL12 NOLS_segment_number: 2 NOLS_segments_positions: 1-20, 165-213 NOLS_segments: MGRNKKKKRDGDDRRPRLVL, TASLHAHSRKKVKRRLTGKARHSGE

FULL	Same as MEDIUM plus the predictor score for each residue in the sequence	> NOL12 NOLS_segment_number: 2 NOLS_segments_positions: 1-20, 165-213 NOLS_segments: MGRNKKKKRDGDDRRPRLVL, TASLHAHSRKKVKRRLTGKARHSGE 0.87 0.79 0.69 ...

COMPLETE	Same as FULL plus the input sequences	> NOL12 MGRNKKKKRDGDDRRPRLVLSFDEEKRREYLTGFHKRKVERKKAAIEEIKQRLKEEQRKLREERHQEYLKMLAEREEALE... NOLS_segment_number: 2 NOLS_segments_positions: 1-20, 165-213 NOLS_segments: MGRNKKKKRDGDDRRPRLVL, TASLHAHSRKKVKRRLTGKARHSGE 0.87 0.79 0.69 ...

^a ^The sequences and scores are truncated in the Table for clarity of presentation.

Finally, for users preferring to run and visualize their predictions locally, there is a virtual appliance version of NoD, which can easily be deployed on a variety of operating systems by a non-specialist user. The virtual appliance version of NoD offers the same functionality as our public server, with the exception of JPred predictions. However, in the near future we intend to release a version which will support JPred.

## Results and Discussion

### Prediction of NoLSs in non-human eukaryotes

Because more NoLSs have been reported in human than in all other organisms combined, the NoLS predictor was originally trained and tested only on human sequences [10]. More precisely, as described previously [10], the predictor was trained on a manually curated positive set of 46 human experimentally validated NoLSs and a negative set consisting of several hundred human proteins chosen because they are believed not to localize to the nucleolus. After training, ten of the NoLS predictions were chosen for experimental validation and all were confirmed as positives [10].

However, the prediction of NoLSs is relevant in all eukaryotes and in particular in their viruses, many of which encode proteins that localize to the nucleolus of their host cells [13]. To investigate whether the human-trained predictor can be applied to other organisms, we searched the literature to find examples of NoLSs that have been experimentally identified in other organisms. In total, we collated 31 eukaryotic or viral NoLSs (including 6 human NoLSs that had not been used for training or testing previously) which are listed in Table 2, along with the position of the experimentally determined NoLSs. Sequences were filtered to remove redundancy within this dataset and redundancy with the original training set as described previously [10]. The full-length sequences of these NoLS-containing proteins were then passed through the NoLS predictor. As with the original NoLS paper [10], only experimentally validated NoLSs of length less than 50 residues were considered for testing. This focuses the testing on those NoLSs that have been most confidently identified by experiment and reduces the likelihood that we are dealing with signal patches (ie signals formed from residues distant in the primary sequence but that come into close proximity in the folded molecule). We considered NoLSs as correctly-predicted if the region of overlap between the predicted NoLS and the experimentally determined NoLS covered at least 60% of the shortest of the two molecules. In many cases, the predicted NoLS region is entirely contained within the experimentally determined NoLS or vice versa. Details of the predictions, including the position of predicted NoLSs, are given in Table 2 and a summary of the prediction accuracy is given in Table 3.

**Table 2:** Detail of NoD predictions on the multi-organism testing dataset assembled

Organism	Protein Accession	Name	Experimentally determined NoLS position	NoD prediction	Ref^a^
Homo sapiens	NP_001012333	Midkine	129-143	120-143	[19]

Homo sapiens	NP_055701	NSA2	10-41	no NoLS	[20]

Homo sapiens	NP_055701	NSA2	131-154	133-155	[20]

Homo sapiens	NP_872604	RASSF5	51-100	78-98	[21]

Homo sapiens	NP_037541	follistatin	93-116 *^b^*	98-121	[22]

Homo sapiens	CAA41051	histone H2B	28-35	15-42	[23]

Mus musculus	NP_001012495	Cxcl12	98-118	92-119	[24]

Mus musculus	NP_081208	NoBP	220-262	230-255 and 276-306	[25]

Mus musculus	NP_082355	aminopeptidase O	688-725	682-712	[26]

Dictyostelium discoideum	XP_002649205	eIF6	31-64	27-49	[27]

Dictyostelium discoideum	XP_002649205	eIF6	246-252	295-320	[27]

Aplysia kurodai	B0FRH7	ApLLP	1-19	1-21	[28]

Aplysia kurodai	B0FRH7	ApLLP	90-120	96-120	[28]

Trypanosome brucei	CAD21884	ESAG8	48-79	no NoLS	[14]

Trypanosome cruzi	XP_817097	Met-III	1-19	No NoLS	[29]

Trypanosome cruzi	XP_817097	Met-III	146-191	No NoLS	[29]

Solanum lycopersicum	Q944N1	LHP1	141-171	141-165 and 276-296	[30]

Arabidopsis thaliana	NP_001078269	HMGB1	1-47	22-60	[31]

Bovine herpesvirus 1	CAA90914	BICP27	86-97	75-108	[32]

Human Adenovirus C	YP_001551773	E4orf4	66-75	61-82	[33]

SARS	P59633	Non-structural protein 3b	134-154	No NoLS	[34]

HTLV-1	BAH85789	Tof	71-98	No NoLS	[35]

Human herpes simplex	P08353	Gamma-1 34.5 protein	1-16	1-22	[36]

Human adenovirus 2	P68950	protein VII	93-112	90-117	[15]

African Swine Fever Virus	AAA87288	I14L	1-14	1-26	[37]

PRRSV (porcine)	AAD00244	N protein	41-48	1-21 and 32-59	[38]

Tomato Leaf Curl Java Virus	BAD90868	Capsid protein	1-30	no NoLS	[39]

Potato leafroll virus	P11624	Capsid protein	17-31	10-64	[40]

Marek's disease virus type 1	AAS01627	MEQ protein	62-78	22-47 and 52-81	[41]

Avian Infectious Bronchitis Virus	CAC39307	N protein	71-78	347-377	[42]

Betanodavirus GGNNV	NP_689432	Protein alpha	23-31	10-40	[43]

^a ^Ref: Reference reporting the experimental NoLS identification

^b ^In [22], the NoLS for follistatin is reported at positions 64-87. These correspond to the positions in the protein once the signal peptide has been removed.

**Table 3:** Accuracy of NoD predictions in all organisms investigated

	distinct protein count	NoLS count	TP*^a^*count	FP*^b^*count	Sensitivity	PPV*^c^*	Specificity*^d^*
**A. Eukaryotes**							

**Mammals^e^**	8	9	8	1	0.89	0.89	0.88

H. sapiens	5	6	5	0	0.83	1.0	1.0

M. musculus	3	3	3	1	1.0	0.75	0.67

**Amoeba^e^**	1	2	1	1	0.5	0.5	0.0

Dictyostelium discoideum	1	2	1	1	0.5	0.5	0.0

**Molluscs^e^**	1	2	2	0	1.0	1.0	1.0

A. kurodai	1	2	2	0	1.0	1.0	1.0

**Trypanosomes^e^**	2	3	0	0	0	N/A	1.0

T. brucei	1	1	0	0	0	N/A	1.0

T. cruzi	1	2	0	0	0	N/A	1.0

**Plants^e^**	2	2	2	1	1.0	0.67	0.5

S. lycopersicum	1	1	1	1	1.0	0.50	0.0

A. thaliana	1	1	1	0	1.0	1.0	1.0

**B. Viruses**							

Mammalian host	8	8	6	1	0.75	0.86	0.88

Plant host	2	2	1	0	0.5	1.0	1.0

Avian host	2	2	1	2	0.5	0.33	0.0

Fish host	1	1	1	0	1.0	1.0	1.0

^a ^TP: true positive

^b ^FP: false positive

^c ^PPV: positive predictive value

^d ^The specificity was calculated as the number of proteins considered for which no FP was identified divided by the number of proteins considered (this defines all non NoLS regions as negatives).

^e ^For each of the count columns, the top row of each of the subsections in the Eukaryotes section represents the sum of the rows below it belonging to this subsection.

As shown in Table 3, mammalian NoLSs and their viral counterparts are well predicted, with sensitivity and positive predictive values ranging from 0.75 to 1.0. This is not surprising because of the close evolutionary distance between humans and other mammals and the constant adaptation of their viruses. Amongst the non-mammalian proteins considered, the *Dictyostelium discoideum *protein investigated has two NoLSs, one of which is well-predicted. The NoLS that was not correctly identified consists of only 7 amino acids and is likely too short for the predictor to find. The two mollusc NoLSs are entirely well-predicted but low numbers of examples in this group of organisms prevents robust conclusions. Similarly, plant and plant-infecting virus NoLSs are generally well-predicted but also suffer from small numbers of examples. However, the human-trained predictor is entirely incapable of identifying the NoLSs experimentally detected in trypanosomes. This agrees well with experiments in which the NoLS of a *Trypanosome brucei *protein, ESAG8, was fused to a fluorescent reporter protein and tested for nucleolar localization in human cells. With an intact trypanosome NoLS, the fusion protein was found to be nuclear but not nucleolar in human cells [14]. This observation and our predictions suggest that nucleolar targeting mechanisms differ significantly between humans and trypanosomes and are not interchangeable. Although a larger sample would be needed to confirm this difference, trypanosomal nucleolar targeting mechanisms might represent good drug targets.

While no experimentally generated negative dataset is available for testing the predictor in non-human organisms, we note that the non-NoLS regions of NoLS-containing proteins provide such a set. For example, the human adenovirus 2 protein VII encodes three basic regions at positions 47-54, 93-112 and 127-141 which represent possible nuclear/nucleolar localization sequences [15]. Deletion constructs demonstrate that only the 93-112 segment targets a reporter protein to the nucleolus [15]. This segment matches very closely the NoD NoLS predictions (see Table 2), providing not only an accurate test example but also perfect negative controls (the two other basic regions are not predicted as NoLSs). Thus, the positive predictive values shown in Table 3 give an indication of the false positive rate of prediction in different organisms. However, while some false positives probably represent prediction errors, others might have occurred because NoLSs were not experimentally mapped with enough precision or more than one NoLS exists in the protein. Larger experimental datasets will undoubtedly help to clarify this problem.

Of the 31 eukaryotic and viral NoLSs considered for independent testing, 22 were correctly identified, resulting in an overall sensitivity of 71%. In addition, 6 non-NoLS regions were also identified as positives (and thus are considered here as false positives) yielding an overall positive predictive value of 79%. Finally, of the 27 proteins considered, 6 were predicted to encode NoLSs in regions not experimentally shown to harbour a NoLS resulting in a specificity of 78% (although we note that some of these false positives might represent NoLSs that have yet to be experimentally identified).

## Conclusions

NoD is currently the only predictor capable of providing and visualizing NoLS predictions for protein sequences.

The web server takes a protein sequence as input and returns the positions and the sequences of the predicted NoLSs. It also draws a graph of the predicted scores for each residue of the sequence.

The command line NoD takes the list of FASTA formatted protein sequences as an input, and for each sequence outputs the number of detected NoLSs, their positions in the full-length sequence and their sequences. However, the command line predictor output is highly customisable and can be adjusted to the user's needs. Finally, the virtual appliance version of the predictor allows the deployment and running of the predictor locally, eliminating data privacy issues.

Cross-species testing shows NoD to perform best for mammalian and mammalian-infecting viral proteins but preliminary results suggest sequences from molluscs, amoebae, plants and their viruses are also well-predicted.

### Availability and requirements

• **Project name: **NoD (Nucleolar localization sequence Detector)

• **Project home page: **http://www.compbio.dundee.ac.uk/nod

• **Operating system(s): **Platform independent

• **Programming language: ***Java*

• **Other requirements: **The command line version requires Java 6 or higher, and the SNNS Batch Interpreter V1.0 [12]. The virtual appliance version requires freely available VMware Player 3.1 [16] or higher, commercial VMware Fusion (for Mac users) [17] or the freely available Oracle VirtualBox v3.2.12 [18]

• **License: **Apache 2

• **Any restrictions to use by non-academics: ***no restrictions*

## Authors' contributions

MSS conceived the project and contributed to its design, carried out the acquisition and analysis of data, contributed to the implementation of the predictor and drafted the manuscript. PVT contributed to the design of the project, the implementation of the predictor and critically revised the manuscript. GJB contributed to the design of the project and critically revised the manuscript. All authors read and approved the final manuscript.

## Acknowledgements and Funding

We would like to thank Dr Tom Walsh for technical expertise. This work was supported by a post-doctoral fellowship from the Caledonian Research Foundation to MSS, by the Scottish Universities Life Sciences Alliance (SULSA), by the European Network of Excellence ENFIN [contract LSHG-CT-2005-518254], and by Wellcome Trust grant WT083481.

## Supplementary Material

Additional file 1 **NoD command line manual**. The additional file describes the usage of the NoD batch predictor command line utility. Click here for file
