# Supplementary material for: NoD: a Nucleolar localization sequence detector for eukaryotic and viral proteins
Source: BMC Bioinformatics. 2011 Aug 3;12:317. doi: 10.1186/1471-2105-12-317 (PMC3166288; doi:10.1186/1471-2105-12-317)
Supplement: Additional file 1 — NoD command line manual. The additional file describes the usage of the NoD batch predictor command line utility. [file 1471-2105-12-317-S1.PDF]

NoD command line manual

NucleOlar localization sequence Detector v. 1.2b (28 April 2011)
http://www.compbio.dundee.ac.uk/nod

```
Usage:
       java -jar clinod-1.2.jar -in=inputFile <OPTIONS>

       -in=<input file>       - required, absolute path to the input file.
                                The input file expected to contain the list of
                                FASTA formatted sequences.

       -out=<output file>     - optional, by default the system prints the
                                output to the console. If the file exists it
                                will be overridden.

       -f=<output format>     - optional, defaults to MEDIUM. Possible values
                                COMPLETE, FULL, MEDIUM, SHORT and MINIMAL (case
                                sensitive)

                                Where MINIMAL - contains the sequence name and
                                the number of NOLS detected.

                                SHORT - same as MINIMAL plus coordinates of
                                the NOLS

                                MEDIUM - same as SHORT plus NOLS reported
                                separately

                                FULL - same as MEDIUM plus scores for each
                                position of the sequence. The scores are
                                reported for every window of size 20 in the full
                                sequence, one score per line. The scores are
                                associate with the first residue of the window.
                                Windows with a score above 0.8 are predicted as
                                NoLSs.

                                COMPLETE - same as FULL plus the original
                                sequence

       -d=<working directory> - optional, defaults to the system temp directory
                              (defined by the "java.io.tmpdir" environmental
                              variable) If the directory does not exist, it
                              will be created. However the parent directory
                              must exist. For example if working directory is
                              set to /home/tmp and home directory does not
                              exist then the execution will be terminated.
                              If the home directory exist but tmp directory
                              does not, the tmp directory will be created.
                              If the /home/tmp directory exist then it will
                              be used. If the /home/tmp directory is not
                              empty, then any files with the same name as
                              generated by the program will be overridden.

       -t=<number of threads> - optional, defaults to the number of cores
                                available on the computer. Maximum number of
                                threads cannot be greater than the number of
                                available cores. If multiple threads are used
                                then the order of the sequences in the output is
                                not the same as in the input.
```

-bm=<batchman path> - *optional, an absolute path to the Batchman executable. By default this path is searched in the environmental variable called BATCHMAN_PATH. If both are provided, the path specified in the command line takes preference. One or the other must be provided.*

-nonols - *optional, suppresses reporting of the proteins with no NOLS detected if specified.*

-clean_sequence - *optional, if specified causes the predictor to automatically remove all non-protein characters from the sequences that contains them.*

The predictor only works with unambiguous protein sequences. Only the following characters considered to be valid: ARNDCQEGHILKMFPSTWYV. If any other character is detected in the sequence the predictor stops the execution and raises the exception unless this flag is specified. In this case, the predictor removes invalid characters from the sequence and writes the information about it into the log file.

-h - *print help (this information). If this flag is given all other options are ignored.*

NolsPredictor requires SNNS Batch Interpreter V1.0 executable which is a part of the Stuttgart Neural Network Simulator (SNNS) v 4.2 software suit available free of charge from http://www.ra.cs.uni-tuebingen.de/SNNS

The path to the batchman executable can be specified either as BATCHMAN_PATH environmental variable or provided as a command line parameter.

Invocation examples:

Example 1:

clinod-1.2.jar -in=inputFile

The predictor loads input from inputFile, prints the predictions to the console, uses system's temporary directory to store the temporary files and uses the number of threads equal to the number of processor cores available on the executing machine. This example assumes that BATCHMAN_PATH environmental variable is defined.

Example 2:

clinod-1.2.jar -in=/homes/input.fs -out=/homes/out.txt -t=1 -d=/homes/tmp -bm=/homes/bin/batchman -nonols -f=MINIMAL

The predictor loads the input from the /homes/input.fs, writes output to /homes/out.txt uses a single thread to perform calculations, writes temporary files to /homes/tmp, uses the Batchman binary from /homes/bin directory, reports results in the MINIMAL format and only for the sequences where at least one NOLS has been detected.

Logging:

NolsPredictor uses Apache log4j library for logging. With its help it is possible to track the execution progress or peer into the details of the

execution of the program which can help to resolve issues. For this log4j should be configured to log all events at the INFO level. Level DEBUG or TRACE can help if you are experiencing problems with the predictor.

Example log4j configuration:

```
logDir = .
log4j.logger.NOD=INFO, ACTIVITY
log4j.appender.ACTIVITY=org.apache.log4j.RollingFileAppender
log4j.appender.ACTIVITY.File=${logDir}/activity.log
log4j.appender.ACTIVITY.MaxFileSize=10MB
log4j.appender.ACTIVITY.MaxBackupIndex=10000
log4j.appender.ACTIVITY.layout=org.apache.log4j.PatternLayout
log4j.appender.ACTIVITY.layout.ConversionPattern=%d{MM-dd@HH:mm:ss} %-
5p %3x - %m%n
```

This configuration creates a log file called activity.log in the current working directory and records all events from the program at the level INFO. The activity file is renamed to activity.1 once it reaches the 10 Mb size and the new activity.log file is created.

To configure log4j, the configuration should be written into the file called log4j.properties and put into the same directory as the program. By default log4j is configured to output only the error messages to the console.

Performance:

NolsPredictor writes 4 temporary files for a single FASTA sequence, therefore for optimal performance the directory for temporary file storage should ideally be located on the local hard drive, as opposed to the remote storage. The predictor throughput rate is about 2 sequences per second on the average computer.
